# The National Insurance Bill

**Published:** 1911-07-29

**Authors:** 


### THE NATIONAL INSURANCE BILL.

### SCOTTISH HOSPITALS MEMORANDUM.

At a meeting of representatives of Voluntary Hospitals, held in Glasgow on July 21, Sir Matthew Arthur, Bart., in the chair, it was agreed that the following memorandum, embodying the views of superintendents and managers of the hospitals and infirmaries of Glasgow and Scotland generally as to the effect of the National Insurance Bill on these institutions, should be forwarded to members of Parliament for their information and guidance with special reference to the amendment of Clauses 8, 15, 16, and 17.

Text of the Memorandum.

The State undertakes to give free medical attendance with free medicine to all insured persons, but no provision is made in the Bill for institutional treatment except for tuberculosis cases in Sanatoria. In order to accomplish this, premiums must be paid by the employer, the employee, and the State.

Voluntary hospitals are now supported by (*a*) legacies, (*b*) donations, and (*c*) annual subscriptions. The annual subscriptions are derived from three main sources—the large employer of labour subscribes a substantial sum either personally or through his firm, the business man and householder give an annual subscription, and the employee subscribes a small sum, usually 1d. weekly. If the Bill is passed in its present form, the employer will not feel disposed to subscribe, seeing he must pay 3d. a week for each employee in his service in addition to his insurance premium under the Workmen's Compensation Act; the general subscriber will feel that if he is compelled to pay 13s. a year for the insurance of each of his domestic servants, he must withdraw his subscription to the voluntary hospital; and the workman himself cannot afford to have an extra 1d. deducted from his wages. Voluntary hospitals will therefore be affected in at least three directions :—

1. Money which in the past has been given voluntarily for the support of these institutions will be diverted into another channel, and no provision is made in the Bill for voluntary hospitals participating in the benefits which will result from the passing of the Bill.

2. Hospitals will have to pay a considerable sum annually in compulsory insurance for their nurses, clerks, porters, scrubbers, servants, etc. (It should be noted in this connection that every hospital treats its sick employees, and in most cases pays them during the time they are sick.)

3. Unless special grants are given from the Treasury, or provision is made in the Bill to recoup the voluntary hospitals for the expense which they will incur for the treatment of insured persons under the Bill, the work of these institutions will be seriously curtailed, and, as a result, medical education will suffer. Up till now, the voluntary hospitals have been exclusively the training schools for medical students as well as the centres for medical research. The voluntary hospitals cannot be done without, and if the funds for their upkeep are not to be subscribed voluntarily, then the State must interpose.

Some Important Data.

The following points are worthy of careful consideration :—

1. Hospitals should be exempted from payment under the scheme for their nurses and staff.
2. Clauses 8, 15, and 16 should be amended to admit of voluntary hospitals participating, and Clause 17 should be deleted.

In the memorandum sent to Principal Sir Donald MacAlister, President of the General Medical Council, by the Chancellor of the Exchequer, it is stated :—

"It is not proposed to make provision in the Bill for institutional treatment and operations."

What then is to be done with insured persons who may meet with a serious accident, or require a serious surgical operation performed, or who require treatment by a specialist and skilled nursing? Are these people to be thrown on charity when they require this institutional treatment which can only be got in the voluntary hospital? These cases cannot be treated satisfactorily in their own homes by the general practitioner.

It is calculated that the voluntary hospitals of the United Kingdom cost nearly £4,000,000 per annum for upkeep. The problem which must be solved by the managers and trustees of these institutions is—How can we continue to carry on these institutions unless the funds are forthcoming? The voluntary principle does not necessarily need to be sacrificed, provided Clause 8 and Clause 15 of the Bill are amended and Clause 17 cancelled, and such other amendments made on the Bill as would adequately meet the points to which attention has been directed in this memorandum. Clause 17 only makes it lawful for an approved society to grant such subscriptions as it may think fit and subject to any conditions they may impose. The funds necessary for the upkeep of these institutions should not be dependent on the arbitrary decisions of approved societies, but should be provided by the Treasury directly or through the Local Health Committees, on which latter the managers of voluntary hospitals should have representation. Further, as in the case of Sanatoria, the contributions of Local Health Committees, and the conditions attached thereto, should be subject to the approval of the Insurance Commissioners.

